# The Subventricular Zone in Glioblastoma: Genesis, Maintenance, and Modeling

**DOI:** 10.3389/fonc.2022.790976

**Published:** 2022-03-10

**Authors:** Jamison Beiriger, Ahmed Habib, Nicolina Jovanovich, Chowdari V. Kodavali, Lincoln Edwards, Nduka Amankulor, Pascal O. Zinn

**Affiliations:** ^1^ Department of Neurosurgery, University of Pittsburgh Medical Center, Pittsburgh, PA, United States; ^2^ Hillman Cancer Center, University of Pittsburgh Medical Center, Pittsburgh PA, United States

**Keywords:** SVZ, glioblastoma, modeling, ventricular, organoid

## Abstract

Glioblastoma (GBM) is a malignant tumor with a median survival rate of 15-16 months with standard care; however, cases of successful treatment offer hope that an enhanced understanding of the pathology will improve the prognosis. The cell of origin in GBM remains controversial. Recent evidence has implicated stem cells as cells of origin in many cancers. Neural stem/precursor cells (NSCs) are being evaluated as potential initiators of GBM tumorigenesis. The NSCs in the subventricular zone (SVZ) have demonstrated similar molecular profiles and share several distinctive characteristics to proliferative glioblastoma stem cells (GSCs) in GBM. Genomic and proteomic studies comparing the SVZ and GBM support the hypothesis that the tumor cells and SVZ cells are related. Animal models corroborate this connection, demonstrating migratory patterns from the SVZ to the tumor. Along with laboratory and animal research, clinical studies have demonstrated improved progression-free survival in patients with GBM after radiation to the ipsilateral SVZ. Additionally, key genetic mutations in GBM for the most part carry regulatory roles in the SVZ as well. An exciting avenue towards SVZ modeling and determining its role in gliomagenesis in the human context is human brain organoids. Here we comprehensively discuss and review the role of the SVZ in GBM genesis, maintenance, and modeling.

## Introduction

Glioblastoma (GBM) is the most common and most aggressive malignant glial tumor found in adults (1, 2). While prognosis varies with factors such as age and specific mutations (2–4), GBM remains an incurable tumor with a median survival of 9 months without treatment and 15-16 months with treatment (5–7). However, a small percentage of patients achieve long-term survival (>2.5yrs) (8, 9). Cases of longer-term survival and response to treatment provide hope that increasing knowledge of the disease pathology can lead to treatments with improved survival. Conventional treatment for GBM includes surgical resection followed by concurrent radiotherapy and temozolomide (TMZ) and subsequently, 6-12 cycles of TMZ (6, 10, 11). Aggressive tumor cell migration and growth preclude complete surgical resection, resulting in a near 100% relapse rate (12–15). Incomplete resection, post-operative recovery time, and neurologic deficits may delay subsequent treatment, thus, leading to GBM progression early within weeks of surgery or delayed within 2 years for a majority of patients (16, 17).

Cancer stem-like cells have been suggested as the origin of many cancers (18). Neural stem/precursor cells (NSCs), in particular, have been linked to cancer (19). Molecular evidence establishes a strong link between stem cells and cancer stem cells (20). Animal studies have corroborated this link, supporting a hypothesis of tumor origination from neural precursor cells (19, 21–23). Furthermore, a clinical report of neural precursor transplantation leading to the formation of donor cell-derived tumors demonstrates a possible stem cell origin of cancer in humans (24). Altogether, these findings provide strong support for NSCs as one of the cells of origin for cancer. Specifically, NSCs in the subventricular zone are implicated (25).

The subventricular zone (SVZ) is a 3-5mm layer between the lateral ventricle, corpus callosum, and striatum (26–28) that harbors the largest population of NSCs in the brain (3, 4, 29–32). The SVZ in humans is characterized by an astrocytic ribbon that is separated from a layer of ependymal cells by a hypocellular layer (33). The SVZ in animals differs in cellular composition and structure from the SVZ in humans (33–35).

Disease modeling towards identifying specific therapies for numerous cancers has been described (36). While numerous models have been developed for GBM research, faithfully recapitulating the microenvironment, structure, and molecular characteristics (36), GBM modeling has remained a challenge. Each model is unique and complex with benefits and drawbacks. Models range from *in vitro* cellular tumor systems to animal models (36). The recent advent of 3D models has increased the ability to effectively model the brain and associated tumors in the human context (37). These models more effectively simulate and maintain tumor structure compared to 2D models. However, challenges remain to model GBM, those include a lack of regionalized organoids and the underdevelopment of an immunological/inflammatory response, as well as the presence of only primitive vascular systems (37). It follows that utilizing a combination of models may be most apt for developing novel and effective therapeutic interventions.

Recent research has shed new light on the role of the SVZ in GBM (38, 39). This review addresses current hypotheses in SVZ involvement in gliomagenesis, maintenance, and modeling standards, as well as the capacity of current models to incorporate these hypotheses.

## Glioblastoma Cell of Origin

Cancer cells expressing stem cell surface markers reside in brain tumors, comprising between < 1% of cancer cells in low-grade tumors and over 25% of cancer cells in high-grade tumors (40, 41). A connection between glioblastoma initiating cells (GICs) and NSCs has been identified, but the specific lineages downstream of GICs remain understudied (42). The GBM stem cell and the astrocyte dedifferentiation theory are the two prevailing hypotheses for the origin of GBM (43, 44).

Both of these theories serve to explain the presence of cancer stem cells within the tumor (45, 46). The astrocyte dedifferentiation theory relies on the multi-step process of tumorigenesis leading a mature astrocyte to dedifferentiate to become a malignant stem-like cell. This model is supported by recent experiments demonstrating the formation of tumors that are histologically similar to GBM after activation of oncogenes and suppression of tumor suppressor genes in astrocytes (47). These experiments show that genetic manipulation of astrocytes can lead to tumorigenesis. To induce GBM formation, both tumor suppressors and oncogenes must be manipulated in astrocytes, whereas progenitor cells only require oncogene activation (44, 47). This manipulation in astrocytes results in their acquisition of stem cell-like characteristics, offering one possible explanation for the similarities between GICs and stem cells (48, 49).

The glioblastoma stem cell theory proposes that GICs are derived from NSCs. NSCs are self-renewing, multipotent cells in the brain responsible for differentiating into neurons, astrocytes, and oligodendrocytes (50–52). These cells are most active during development; however, recent evidence has suggested small populations in specific stem-cell niches remain functional in the adult brain (53–57). As such, neurogenesis in the human brain continues throughout life (58, 59). The glioblastoma stem cell theory is based on a longstanding hypothesis that cancers arise from a stem-like cell population, and thus, that tumors contain a subset of multipotent cells with stem cell characteristics. In the case of GBM, partially differentiated glial cells including oligodendrocyte precursor cells (OPCs) and astrocyte precursor cells may contribute to or be responsible for tumorigenesis (38). Lee et al (38) proposed that the most likely pathogenesis involves driver mutations in NSCs that contribute to tumorigenesis after differentiation into the oligodendrocyte cell line. The presence of cells with stem cell-like characteristics has been identified in many cancers (60), including brain tumors (41, 44, 61). Clinical evidence supporting this theory includes the formation of a donor-derived brain tumor after NSCs were injected intracerebrally and intrathecally into an Ataxia Telangiectasia patient (24). This case example demonstrates a stem cell to tumor transition in the human brain. Considering the heterogeneity of GBM, each of these theories may contribute a portion of the total tumor population included in the category of GBM.

## Neural Stem Cells and Glioblastoma Stem Cells Are Molecularly Related

Many molecular characteristics are shared between GBM stem cells (GSCs) and NSCs. Genome-Wide CRISPR-Cas9 screens of NSCs and GSCs identified several genetic commonalities (20). SOCS3, a modulatory protein that is responsible for maintaining stemness in NSCs (62), was identified as a top-scoring GBM-specific fitness gene (20). Loss of function of SOCS3 leads to downregulation of multiple GSC fitness genes, upregulation of neuronal progenitor markers, and ultimately GSC differentiation (20). SOX2, another important NSC factor (63), is also a high-scoring fitness gene for both NSCs and GBM (20). Other genes with similar fitness scores in NSCs and GSCs include SQLE, CDK6, and DOT1L (20). Similar fitness scores in these genes provide evidence that developmental growth patterns are reactivated in GBM (20). Some genes with high fitness scores in GBM had low fitness scores in NSCs, including JUN and SOX9 (20). This could suggest that GBM-specific gene activation promotes the maintenance of GSCs (20). Stem cell gene networks, similar to those of non-transformed NSCs, generate and maintain GSCs (20).

Proteomic analysis similarly highlights the relationship between NSCs and GSCs (64–71). Of 108 proteins differentially expressed in GSC, NSC, and other tumor tissues, 22 were overexpressed in GSC and NSC but not tumor tissue. Most of these genes are involved in chromatin, mRNA, and DNA processing (64). Pathways necessary for self-renewal properties are common between NSCs and GSCs (66–72). One of the proteins overexpressed in GSCs and NSCs is vimentin (64, 73). Hepatoma-derived growth factor (HDGF), an angiogenesis-promoting factor, is expressed at normal levels in NSCs but is overexpressed and secreted in GSCs (64), indicating a potential oncogenic alteration of a normal NSC process. Overexpression of HDGF is implicated in various cancers (74–80) including GBM (64). In addition to HDGF, other growth factors associated with development are produced in GSCs including vascular endothelial growth factor (VEGF), basic fibroblast growth factor (bFGF), transforming growth factor-alpha (TGFα), and stromal-derived factor 1 (SDF1) (64, 81–84). A study specifically examining chromosome 19 proteins in GSCs found upregulation of multiple molecular patterns related to stemness and development (85). These molecular markers highlight the relationship between NSCs and GSCs, as well as the potential avenues for the transformation of NSCs to GSCs.

## Subventricular Zone

### Human Subventricular Zone

**Figure 1:**
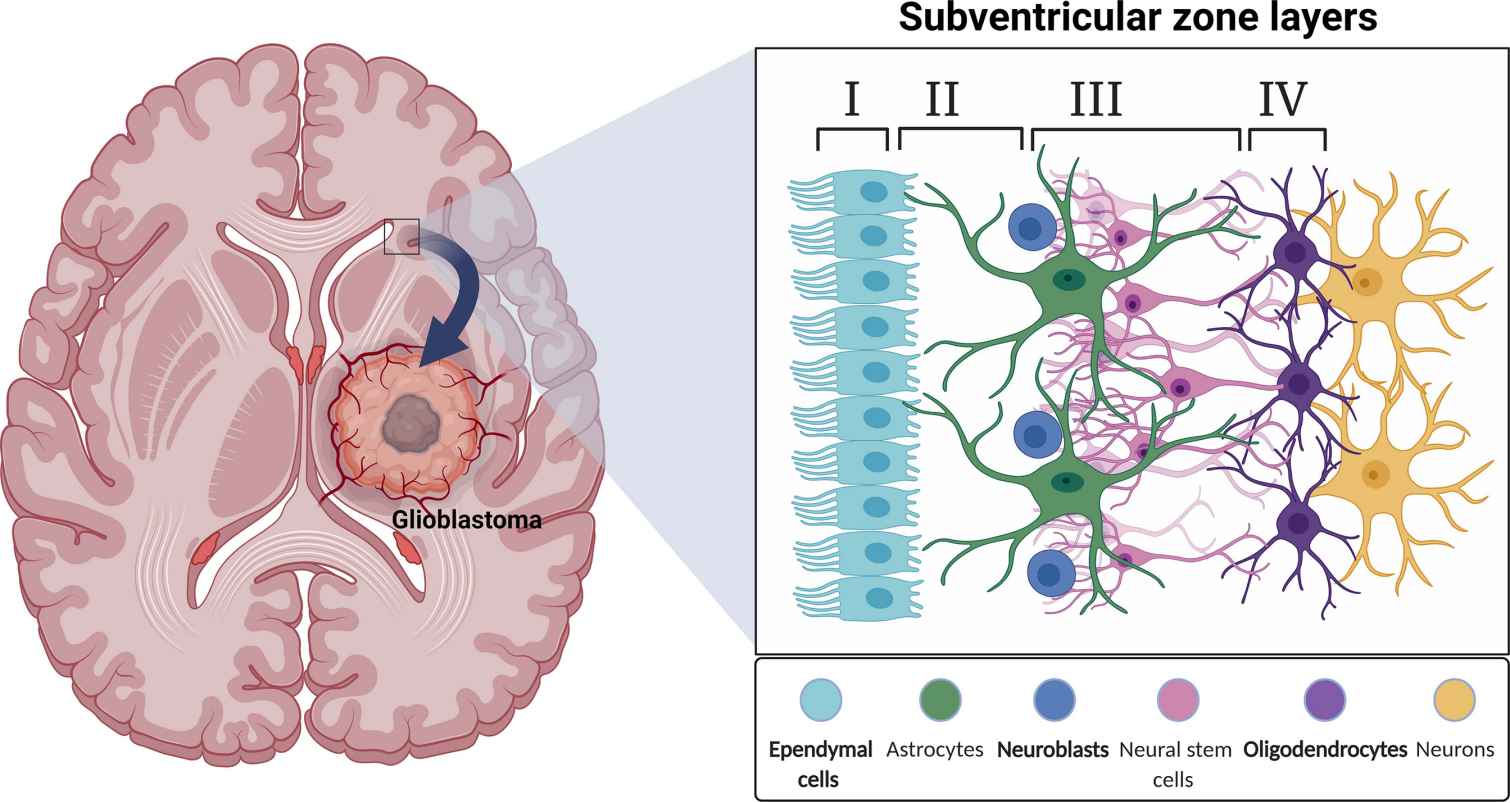
A schematic illustration depicting a coronal view of the subventricular zone (SVZ) neurogenic niche in the adult human brain.

The SVZ is the largest neural stem cell niche in the adult brain. In humans, the SVZ is divided into four regions comprised of different cell types. The ependymal layer (Layer I) is the cellular layer closest to the ventricle common to all areas of SVZ within the brain. The hypocellular layer (Layer II) borders the ependymal layer and consists of basal processes of ependymal cells, astrocyte processes, and diffuse astrocyte cell bodies. Opposite the hypocellular gap from the ependymal layer is the astrocytic ribbon of cells (Layer III). This is the proliferative region where astrocyte-like cells act as stem cells (33, 86). Layer IV is the transitional zone to the brain parenchyma. Myelinated neuronal processes and neuron bodies are abundant in this area (87) (**Figure 1**). The cytoarchitecture of the human SVZ is unlike any other studied mammals. Cell types present in this region include astrocytes, NSCs, neurons, ependymal cells, oligodendrocytes, OPCs, and neuroblasts with transitory amplifying progenitors noticeably missing (39). The human subventricular zone is unique in its organization and cellular composition making it difficult to translate research from animals to humans.

### Subventricular Zone in Humans vs. Subventricular Zone in Animals

Structurally, the SVZ in studied animals differs quite significantly from the human SVZ. The SVZ in rodents is not separated into four distinct layers; in fact, no hypocellular zone exists in adult mice (34). The hypocellular zone is only reported in humans and bovines (88), with all other mammalian models having close contact between ependymal cells and NSCs (35). Another unique characteristic of the human SVZ is the astrocytic ribbon containing proliferative cells previously thought to be NSCs (33, 89). No progenitor cells or migration has been observed from this region, calling into question the activity of these “NSCs” (89). However, when cultured *in vitro*, astrocytes from this region have the capacity to form neurospheres consisting of astrocytes, oligodendrocytes, and neurons (90) potentially indicative of function in the human SVZ. Regardless of function, the astrocytic ribbon is an aspect of the SVZ unique to humans.

Cytoarchitecture also differs. The rodent SVZ includes four cell types based on electron microscopic analysis of ultrastructural characteristics. Unlike in humans, the only cell types are astrocytes, transitory amplifying progenitors (type C cells), neuroblasts, neurons, and ependymal cells (35, 91). NSCs have a radial morphology similar to radial glial cells, their predecessors. NSCs are capable of giving rise to type C cells (29) and ependymal cells (92, 93). Type C cells, located near the blood vessels, are rapidly dividing cells that give rise to OPCs, neuroblasts and astrocytes (94–98). Neuroblasts migrate from the SVZ to the olfactory bulb (OB) where they can undergo neurogenesis (27, 99–101). The rodent SVZ is characterized by a much higher number of neurons than the human SVZ (33, 35, 87). The number of proliferating cells in the human SVZ is also much lower than that seen in rodents (33, 87, 102). While the rodent SVZ has more neuroblasts and increased proliferation, GFAP+, nestin+ radial glia observed in the human third ventricle SVZ are absent from the corresponding third ventricle SVZ in mice (34). Lastly, the rodent SVZ contains chains of migrating neuroblasts, which the human SVZ lacks (103). Migration from the SVZ in adult humans remains controversial (33, 58, 102, 104). In postnatal humans, migration from the SVZ occurs but quickly declines (105). In addition to the rostral migratory stream, a medial migratory stream was identified in humans leading from the SVZ to the prefrontal cortex (58). The medial migratory stream is absent from postnatal mice (58). In rodents, neural progenitor cells differentiate into local interneurons, granule cells, and periglomerular cells after migrating to the OB (106–110). Despite a much lower rate of neurogenesis, the adult human SVZ retains the ability to regenerate neurons (58, 111, 112). Neurogenesis in the SVZ can be regulated by GABAergic, dopaminergic, serotonergic, cholinergic, and nitric oxide-releasing neurons (113–116). Specific circumstances, including depression and Parkinson’s disease, increase neurogenesis from insignificant to noticeable levels (117, 118).

Studies over the past five decades have demonstrated cellular proliferation in the SVZ in multiple species including mice, rats, rabbits, voles, dogs, cows, monkeys, and humans (33, 87, 88, 119–126). Rodent and other model SVZs share characteristics with the human SVZ, yet there are structural and functional differences (127). One major difference is the destination of new neurons from the SVZ. In humans, the rostral migratory stream contains only a small number of migratory neuroblasts that do not form the chains observed in rodents. These neuroblasts express immature neuron and proliferation signals similar to those in rodents but do not migrate to the OB, a major destination for neuroblasts in rodents and monkeys (119). Carbon 14 analysis of cells in the human OB confirms negligible post-developmental neuronal proliferation in this area (128). Rather, the striatum seems a much more likely target for neuroblast migration in humans. Located adjacent to the SVZ, the striatum has cells co-expressing the neuroblast markers DCX and PSA-NCAM, indicating migration to this region (129). Carbon dating of a subpopulation of DARP23-negative interneurons in striatum demonstrates a 2.7% turnover rate, significantly higher than that of the OB (< 1% over 100 years). Furthermore, recently developed striatal neurons and neuroblasts co-express the markers calretinin and neuropeptide Y, supporting an SVZ origin for striatal neurogenesis (129). Other animals demonstrate decreased striatal neurogenesis compared to humans (130–135). Research in mice found Notch-dependent local astrocyte-mediated neurogenesis in the striatum (136). It is unclear the extent to which this occurs in humans (135). These differences highlight the challenges involved with translating animal research to humans in this area.

### Additional Elements of the Subventricular Zone

The following elements of the SVZ have been established in non-human mammals, but the extent to which they are present in the human SVZ is unclear. The SVZ stem cell niche is comprised of various cellular and acellular components along with the major cell types. Blood vessels influence differentiation and migration patterns in this niche. Brain-derived neurotropic factor (BDNF) signaling guides neuroblast migration parallel to blood vessels adjacent to the SVZ (92, 137, 138). The SVZ has a leaky blood-brain barrier, permitting neural progenitor cells (NPCs) of the SVZ to respond to signals in the blood more readily (95). SVZ cells are drawn to blood vessels by molecules secreted by endothelial cells (139). Endothelial cells also promote neuroblast proliferation (94, 95, 140) and influence cell migration to and from the niche through SDF1/CXCR4 signaling (139). An extensive basal lamina contacts nearly every cell in the SVZ providing an avenue for molecular signaling to the entire niche (141, 142). These extracellular matrix structures, called fractones, bind growth factors and modulate NSC proliferation in the SVZ (143). Fractones are fractal-like structures consisting of laminins, collagens II and XVIII, nidogen, HSPGs, and perlecan (143). Laminin constructs both NSC and cancer stem cell (CSC) niches (144, 145) and supports stem cell renewal (146). Additionally, laminin interaction with integrin α-6 is important for the maintenance of NSCs and CSCs (147). Non-stem tumor cells produce laminin α-2, which permits GBM stem cell growth (148).

Macro-structure of the SVZ also facilitates signaling and subsequent cellular responses. Viewed from the ventral surface, the SVZ is organized in pinwheel structural units composed of ependymal cells spiraled around astrocytic processes. Cells in the pinwheels are connected by adherens junctions. Adherens junctions allow one daughter cell to remain a stem cell while the other differentiates into a progenitor. The pinwheel structure is important for stem cell proliferation and is characteristic of other stem cell niches in the body (149).

Gliogenesis is prominent in the developing SVZ from embryonic day (E) 90 until after E125. The SVZ serves as the origin of many of the glial cells in the mammalian brain (150–152). Studies examining multiple sclerosis and the rodent OB indicate that gliogenesis in the SVZ continues in adults (153, 154) and evidence indicates that injury in the adult brain leads to increased gliogenesis from the SVZ (155, 156). Galectin-3 (Gal-3), up-regulated in brain injury, inflammation, and cancer, has a suggested role in modulating both neurogenesis and gliogenesis in the adult SVZ (157).

## Neural Stem Cells in the Subventricular Zone Implication in the Origin of Glioblastoma

### Subventricular Zone Neural Stem Cells Play a Role in Tumorigenesis

Genomic and proteomic analyses of GBM and the SVZ have supported an association between the two. Recent molecular and genetic analysis of human GBM by Lee et al. (38) backs the theory that GBM develops from NSCs in the SVZ. They described direct molecular and genetic evidence from glioblastoma patients’ tissue and mouse models that there were astrocyte-like NSCs in the SVZ that could be the cell of origin. These cells contain the main driver mutations known to form GBM in humans. In their experiment, they performed sequencing of patient-matched tissues types (normal SVZ tissue, tumor tissue, and normal brain cortex or blood) from 28 patients with variable genetic profiles including isocitrate dehydrogenase-1 wild type (IDH-WT). They concluded that low-level driver mutations of GBM were present in the non-tumor SVZ tissue in 56.3% of IDH wild-type patients. Furthermore, single-cell sequencing and laser microdissection analysis of the obtained brain tissue as well as genome editing of their mouse model showed astrocyte-like NSCs carry driver mutations that lead to the development of high-grade gliomas (38). Additionally, extensive analysis of 28 tumors from both adults and children by Neftel et al. (158), indicates the presence of four cellular states that drive GBM malignant cells heterogeneity. These cellular states are associated with cycling cells representing mostly NPC-like and OPC-like states, particularly in pediatric tumors. Earlier reports of lineage tracing methods (159) also revealed significant aberrant growth prior to malignancy in OPCs. These findings suggest OPCs could be the major source of malignancy though initial mutations could occur in NSCs. This highlights the importance of analyzing premalignant stages to identify the cancer cell of origin.

SVZ-related markers, such as GFAP and vimentin, are upregulated in GBM (160). This association supports the hypothesis that tumor cells in GBM are most related to the SVZ cells (160). Specifically, neuroblasts in GBM contain high levels of c-Myc, implicating the population of SVZ cells with high c-Myc expression in oncogenic transformation (160). In fact, overexpression of c-Myc may play a role in tumorigenesis and migration as it is expressed in SVZ cells with migratory potential (160).

Furthermore, restriction of proteases in GBM inhibits tumorigenesis, providing support for the theory of long-distance migration of GBM pathogenesis. Genomic investigation of SVZ-associated GBM supports this analysis, identifying genes commonly altered in SVZ and GBM (161). Differences between SVZ+ (SVZ-associated GBM) and SVZ- (Non-SVZ-associated GBM) GBM have also been observed (161). Notch signaling upregulation in SVZ+ GBM is correlated with Notch upregulation in the SVZ. The differential expression of various Notch signaling molecules is associated with predictable prognostic factors, including overall survival (OS) and progression-free survival (PFS) (162).

Proteome analysis of SVZ+ serum and tissue shows increased acute-phase proteins, lipid carrying proteins, and increased regulatory proteins potentially implicated in increased SVZ+ aggressiveness (163). CD133 expression, which is associated with a shorter time to distant recurrence, is greater in SVZ+ tumors (determined by imaging) than in SVZ- tumors (164). Additionally, the prognosis for GBM is strongly associated with the intracranial location in relation to the SVZ. Tumors contacting the SVZ have worse OS and PFS compared to more distant GBM (165). Furthermore, recurrence of GBM is significantly associated with neurogenic regions (12). The niche factors secreted by the SVZ promote proliferation and migration of GBM progenitor cells, promoting tumor growth and progression (166, 167). In contrast, the hippocampus is often spared from GBM invasion, possibly due to a less compatible extracellular matrix (ECM) (168). Furthermore, NSCs in the hippocampus are less likely involved in tumorigenesis. There are a few factors that differentiate NSCs in the SVZ from hippocampal NSCs in the subgranular zone (SGZ). Like NSCs in the SVZ, hippocampal NSCs have an apical process that contacts blood vessels; however, their basal process contacts neurons and glial cells (169, 170). These stem cells lack CSF contact which is normally a source of factors moderating proliferation for NSCs in the SVZ (25, 171). Abnormal signaling from the CSF is a potential mechanism for malignant transformation (25). Additionally, NSCs in the SGZ only differentiate into local granule neurons. The SGZ niche promotes differentiation without migration, whereas the SVZ promotes proliferation and migration while restricting differentiation (25).

Commonalities of GSCs and NSCs in the SVZ include nestin expression, proliferation capability, high motility, diverse progeny, association with vasculature, and communication with other niche components (25, 172). Much like NSCs (173, 174), GSCs rely on endothelial cells for factors promoting self-renewal, tumorigenicity, and survival (175–177). GSCs are also able to recruit microglia through cytokine production (178–180), which in turn promote tumor growth through angiogenesis and trophic factors (181). Much like in the tumor niche, NSCs and microglia regulate each other in the SVZ (182). Additionally, astrocytes and ECM proteins support the proliferation of both GSCs and NSCs (183–186). These similarities highlight the likeness the tumor niche displays for the SVZ. One notable difference is the lack of CSF within close proximity to cells within the tumor niche, which may contribute to the tumor pathology due to absence of regulation from CSF signaling (25).

Tumorigenesis experimentation in mice supports the theory that GBM-like invasive tumors originate in the SVZ. High-grade tumors are formed from NSCs/NPCs in mouse models after migration. Migration occurs following the leader cell creation of an infiltrative path. Most infiltrations occur along blood vessels, fiber tracts, or over the surface *via* the subarachnoid space (187). In mice, SVZ cell migration occurs through the rostral migratory stream to many areas including the OB, hippocampus, and striatum. These cells have more migratory potential than other NPC niches, traveling further and to more locations (153). Neuroblasts have been identified in high numbers between the SVZ and the tumor in mice models, indicating SVZ cell migration to the tumor. Upregulation of neural precursors in the ipsilateral SVZ in mice with tumors contributes to this hypothesis (188). Follistatin secretion from NPCs decreases tumor growth and can even inhibit tumor growth *in vitro* (189). Follistatin expression by NPCs in the SVZ may explain why migration occurs before tumorigenesis. Neural precursor cell migration from the SVZ to the tumor zone is a critical finding in the pathogenesis of GBM tumors. This pathway represents an important target of future therapy and more models are needed to further investigate this relationship.

### Common Genes Implicated in Glioblastoma Play a Role in the Subventricular Zone

There are several common mutations associated with human GBM. Primary GBM is classified by *de novo* mutations without evidence of a prior lesion (160). Primary GBM typically results from epidermal growth factor receptor (EGFR) amplification and loss of PTEN (190), while secondary GBMs result from IDH1 or IDH2 mutations (191, 192). Inactivation of TP53 (23), PTEN (193), and mutations in telomerase reverse transcriptase (TERT) (194, 195) are also commonly thought to contribute to the pathogenesis of GBM. Each of these genes, with the exception of IDH1, is known to be involved in the control of the SVZ NSCs (160). Matarredona and Pastor (25) recently reviewed some of the most common genetic mutations and their involvement in implicating the SVZ in GBM development.

Epidermal Growth Factor (EGF) induces proliferation and inhibits differentiation of NSCs in the SVZ (196–198). Amplification of the EGFR gene has been proposed as a potential mechanism for the development of GBM because of its role in the SVZ (160, 199). Both TP53 and PTEN are tumor suppressor genes. TP53, which modulates cell division, differentiation, and proliferation in the SVZ, is commonly mutated in both primary and secondary GBM (23, 190, 200–202). PTEN is involved in regulating migration, apoptosis, and proliferation for NSCs in the SVZ (203, 204). Knockout of TP53 or PTEN induces proclivity towards oncogenic transformation (193, 205). In adult mammals, telomerase expression is restricted to the OB and the SVZ (206), where it permits the growth and survival of NSCs (207). TERT is frequently upregulated in cancers (208), including more than half of GBMs (194, 209). In a study of human GBM mutations in IDH1 wild-type, the tumor-free SVZ had TERT promoter mutations, suggesting this could be an early mutation in the progression from NSC to GBM (38, 194).

While IDH1 has no known direct influence on the SVZ, IDH1 mutation is correlated with platelet-derived growth factor (PDGF) expression in GBM (210). PDGF promotes the proliferation of NSCs in the SVZ (211). Some other factors and pathways commonly altered in GBM and SVZ include c-Met, FoxO3, the Wnt pathway, and the sonic hedgehog pathway (160, 172, 212–216). These mutations provide strong evidence that SVZ NSCs are the origin of GBM in humans and accentuate pathways that could be targeted with therapeutics.

### Clinical Significance of the Subventricular Zone in Glioblastoma

Understanding the role of the SVZ in GBM provides significant clinical value. Proposed therapy for GBM includes administering radiation to the SVZ to prevent tumor reoccurrence. So far, the reported effect of SVZ irradiation on outcomes in GBM patients has been inconsistent. In a meta-analysis of four studies observing the effects of high vs lose dose radiation on prognosis, increased radiation dose to the ipsilateral SVZ significantly increased PFS while failing to significantly improve OS (217). Irradiation dose to the contralateral SVZ did not significantly improve PFS. Contralateral SVZ radiation dose effect on OS was not analyzed as it did not meet the study’s inclusion criteria. Higher cutoffs for “high dose irradiation” in the various studies correlated with increased PFS and OS as compared to lower cutoffs (217). Gupta et al. (218) found increased OS with increased radiation to the ipsilateral SVZ but decreased OS with increased radiation to the contralateral SVZ. Consistent with the results described above, Rizzo et al. (219) showed that increasing bilateral SVZ radiation dose directly correlates with increased PFS and OS. Furthermore, they found that a high radiation dose to the ipsilateral SVZ is associated with increased PFS and that there is no correlation between the dose administered to the contralateral SVZ and OS or PFS (219).

**Figure 2:**
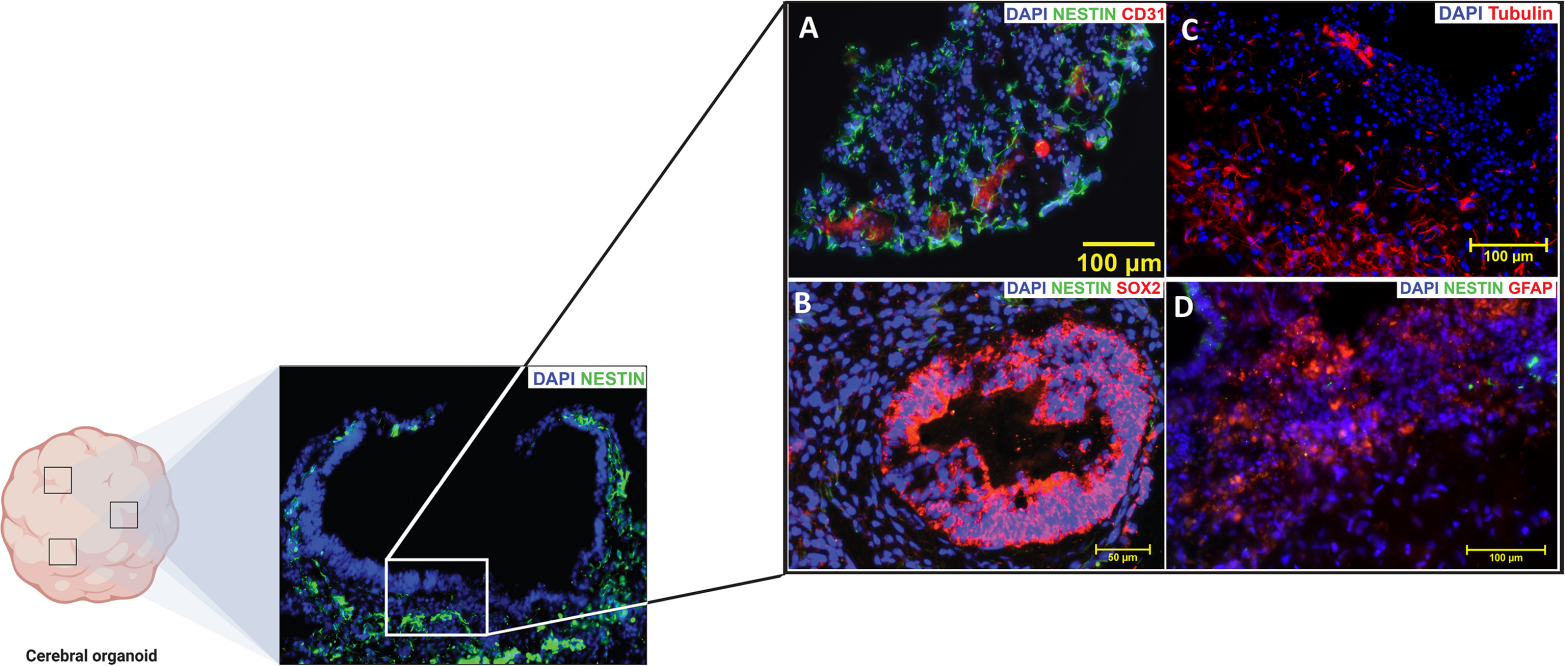
The subventricular zone (SVZ) of a mature cortical organoid highly resembles the (SVZ) of an adult human brain. Positive immunofluorescence signatures of the common (SVZ) markers are represented in **(A–D)**. NESTIN (Green) is an intermediate filament protein that is expressed by neural stem cells (NSCs) in the subventricular zone (SVZ) and it is generally recognized as a marker of undifferentiated nervous system cells. CD31 [Red, **(A)**] also known as Platelet endothelial cell adhesion molecule-1 (PECAM-1), is a glycoprotein highly expressed on endothelial cells and it is generally recognized as blood vessel markers. SOX-2 [Red, **(B)**] is an HMG-Box transcription factor that is expressed in neural progenitor cells and is considered as a marker of high pluripotency. Beta-Tubulin III [Red, **(C)**] an essential structural protein of the neural microtubule network that correlates with the earliest phases of neuronal differentiation. GFAP [Red, **(D)**] is a marker for the glial fibrillary acidic protein expressed by astrocytes and ependymal cells during development. (Source Zinn lab).

Gross total resection (GTR) may account for some of the variations in the results, as Chen et al. (220) found that high dose irradiation of the SVZ improves PFS only in patients with GTR, not in patients with subtotal resection or biopsy. In patients with GTR, PFS and OS were both improved with high-dose irradiation when compared to low-dose irradiation. This suggests that residual tumors may be responsible for recurrence in irradiated patients without GTR, and therefore, that irradiation may potentially prevent GBM recurrence originating from the SVZ (220). CXCL12 mediated upregulation of mesenchymal traits protects GSCs located in the SVZ from radiation, potentially explaining the increased effectiveness of higher doses of radiation (221). These data point to promising evidence that links radiation of areas of the SVZ to increased measures of survival and highlight the importance of studying GBM in the context of the SVZ. By means of human cerebral organoid SVZ models, we hope to expand on the research that has highlighted this relationship between the SVZ and GBM prognosis (**Figure 2**).

## Models

There are several different methods of modeling GBM. Robertson et al. (36) organize GBM models into five separate categories: Patient-derived glioblastoma cell lines are cultured directly from patient tumors; engineered GBM-like cell lines are cells genetically manipulated to represent GBM; *ex vivo* models study animal tissue in relation to GBM; *in vivo* tumor transplantation models involve human tumor implantation in animal models, and genetically engineered mouse models (GEMMs) involve the introduction of GBM through germline genetic manipulation. Organoid models are 3D patient-derived suspensions discussed separately. Each model has unique benefits and drawbacks in the study of GBM.

### Patient-Derived Glioblastoma Cell Lines

*In vitro* models are often the simplest models. They involve culturing and experimenting on cell lines meant to represent GBM. Investigators have the choice of experimenting on widely used highly passaged ‘classic’ cell lines or patient-derived models such as low passage primary cell lines or tumor tissue slice cultures. The reliability of classically used cell lines, and the tumors they produce, have been called into question because they have assumed a differentiated state that may no longer reflect the GSCs intended to be represented. Tumors resulting from orthotopic transplantation of the classic cell lines that are grown in serum adherent to plates in 2D culture settings, often do not resemble GBM, but rather grow as an encapsulated tumor in the mouse brain, resembling the growth pattern of brain metastasis (65). One of the most popularly studied cell lines, U87MG, is genetically different in many respects and no longer matches the original culture (222, 223). Even without these issues, *in vitro* cell models using classic cell lines, have limited utility for simple mechanistic studies and likely should be moved away from (36, 222, 224).

Unlike classic cell lines, patient-derived GBM cell models retain the genetic and transcriptional state of the parent tumor, when cultured in similar conditions, long term (41, 61, 65, 225, 226). Cultures are deemed viable for long-term self-renewal after 10 passages which equate to at least 2 months in culture and can be frozen for long-term preservation (227). In one study, eight out of nine patient-derived models that lasted at least 8 passages were still viable after 50 passages (228). However, in practice low passage cultures are always archived. Patient-derived GBM cell models were originally grown as neurospheres in suspension culture, but the suspension is not necessary for survival and expansion. Adherent cultures are viable, permitting easier experimentation with these models (147, 229, 230). Additionally, adherent cultures were effective in deriving new cell lines in >90% of cases when using IDH-WT GBM cell lines (36, 224, 226). While adherent cultures are often easier to manipulate and study, suspension cultures can be developed into organoid models. These models are discussed more in a later section.

### Engineered Glioblastoma-Like Cell Lines

GBM can also be engineered *in vitro*. Introduction of driver mutations stepwise into NSCs or other cells, *in vitro*, can cause these cells to transform phenotypically into cancer cells. They can then be transplanted and studied *in vivo* (36). Various methods such as plasmid transfection, lentiviral or retroviral transduction (48), and CRISPR/Cas9 technology (231) are used to induce these mutations and produce a GBM-like phenotype. CRISPR/Cas9 technology enables gene knock-out/in and more precise insertions/deletions compared to previous methods. It also allows for more experimental control through novel genetic screening techniques *in vitro* and *in vivo* (232, 233). CRISPR is effective for genetic manipulation of both human and mouse NSCs and could be used in a variety of future experiments towards this (36, 234). Additionally, it has been used to induce tumorigenesis in organoid models by knocking out tumor suppressor genes. The resulting tumor cells are molecularly similar to GBM and can be implanted into mice to grow into tumors (235, 236).

### *Ex Vivo* Animal Models

*Ex vivo* modeling is a popular neuroscience procedure adapted for GBM study (36, 237). Mice are the most common model due to their short breeding times, relatively low cost, easy genetic manipulation, and mammalian organ systems (36). Both slice culture methods and whole animal models can be studied. Slice culture methods allow an accurate microanatomical analysis of tissue-tumor interaction, which has provided insight into the interaction between GBM and the SVZ (238). However, whole animal models are often necessary as comprehensive disease-relevant models (36). Both tumor cell transplantation and genetically manipulated *de novo* tumors are viable methods of tumor induction of GBM *in vivo*.

### *In Vivo* Tumor Transplantation Models

Tumor cells are directly transplanted into the brain or the skin in *in vivo* tumor transplantation models *via* orthotopic or subcutaneous injections. Orthotopic grafts are preferred due to spatial and temporal selectivity which allows for the induction of similar tumor physiology in multiple mice (36). Some drawbacks of orthotopic grafts include the technical challenge of implantation, lack of control over engraftment and seeding, and disruption of normal tissue architecture where the injury is caused by the injection procedure. Subcutaneous grafts are easier to introduce but lack the specific brain microenvironment and infiltration characteristic of GBM (239). Patient-derived orthotopic xenografts (PDOX) models involve the implantation of human tumor cells into immunocompromised mice, potentially simulating the tumor microenvironment of human GBM (36). Golebiewska et al. (240) reported the generation of a unique set of organoids and patient-derived xenografts of various glioma subtypes and corresponding longitudinal PDOX from primary and recurrent tumors. The model they presented captured a wide spectrum of the molecular genotypes of GBM that highlights the potential of these models for precision medicine. The mutations described in their models include: IDH1, ATRX, TP53, MDM2/4, amplification of EGFR, PDGFRA, MET, CDK4/6, MDM2/4, and of CDKN2A/B deletion, PTCH, and PTEN. With regards to the corresponding PDOX model, they found that it recapitulates the limited genetic evolution of gliomas in patients following treatment. The model they presented showed a clinically relent response to TMZ and targeted therapies and could be used as starting point to develop more advanced models that may help develop a therapeutically effective GBM precision treatment modality.

### Genetically Engineered Mouse Models

GEMMs are created by introducing germline genetic mutations of tumor suppressors and oncogenes. This can occur *via* mutagen exposure (241), Cre-lox recombination (19), lentivirus administration (242), or CRISPR- technology (36, 243). Selective breeding allows for the maintenance of mice litters with reproducible, mutated genotypes that are more susceptible to developing tumors and are useful for experimentation (36). These models provide insight into initiation events and driver mutations for GBM. A GEMM study revealed NF1 as a driver mutation for malignant astrocytoma (244), contributing to the subsequent discovery of NF1 as a driver mutation in GBM (245). Other work with GEMMs supports the theory that GBM derives from NSCs in the SVZ (242, 246–248). These studies found that NSCs are easier to transform to tumor cells than astrocytes are (242, 246, 248) and that expression of an IDH1 mutation in the adult SVZ can model gliomagenesis (247).

### Organoids

Three-dimensional organoid models are some of the most useful models for studying GBM. Lancaster et al. (249) developed the first organoid model in 2013 by creating neuroectoderm tissue from induced pluripotent stem cells (iPSCs) and then suspending this tissue in a rotating bioreactor to enhance growth. These models recapitulate the cellular heterogeneity and structure seen *in vivo* (249). Organoids can either represent a brain structure or a tumor cytoarchitecture by culturing iPSCs or GSCs respectively (250). Eventually, the model develops to contain differentiated cells of various populations, mirroring the microenvironment of the brain, or tumor, structure. Organoid models allow cells representing a large spectrum of differentiation to coexist within a model (250). Additionally, primary tumors are able to grow to size in organoid models allowing expression of necrotic and hypoxic features of human tumors (250). These features can create a relatively realistic experimental model of a tumor in the human context; however, there are limitations to this model system. Cerebral organoid models take months to culture and can be highly variable, as well as often lack functional vasculature or immune responses (36). Despite these drawbacks, we believe human cerebral organoid technology is an excellent adjunct model system to the current models described above. Other models remain important as adherent cultures are used when a more reductionist model is needed and suspension cultures are used when a more comprehensive heterogenic model is necessary.

Azzarelli et al. (37), identified five different methods developed to create organoid models: (1) adding minced GBM specimen to Matrigel (250), (2) culturing extracted tumor into a matrigel-free serum-free environment on an orbital shaker (251), (3) nucleofecting embryonic stem cell brain organoids (235, 236), (4) adding 2D cultured patient-derived GSCs to embryonic stem cell brain organoids (236, 252), (5) and 3D bioprinting of GBM and endothelial cells with added ECM components (253). Preference for 3D organoid models stems from their ability to potentially recapitulate *in vivo* response to therapy more accurately than other models such as 2D cultures and PDOXs (235, 250, 252). Additionally, organoids are advantageous because they are able to culture a heterogeneous population of cells in the same environment (37). This allows CSC heterogeneity and development to be studied in the proper environment surrounded by a heterogeneous population of cells (37, 254). Recent techniques have enabled the creation of organoid models within 1-2 weeks (251), dramatically reducing the previous procedural time of multiple months (235, 236, 250, 252). This time reduction is key for therapeutic relevance because patients may begin treatment 1-2 weeks after surgical resection (37).

Some future challenges for 3D models include maintaining tumor complexity, establishing a microenvironment to mimic inflammatory responses, and reducing variability (255). A combination of models that takes advantage of each model’s strengths may be most beneficial (37). In some cases, tumors were unable to develop despite genetic alterations consistent with pathogenesis (255). Regionalized organoids for the area of origin of a particular tumor may be required to solve this problem (255–258). It follows, that if human cerebral organoids do demonstrate a relatively faithful microarchitecture and presence of various differentiated cell types, that organoids may be an ideal model to study the human SVZ and how it relates to gliomagenesis.

### Subventricular Zone Organoid Models

Organoid models have a variety of uses at various stages of research. Kim et al (259) reasons that organoid research is useful in four capacities: basic research, biobanking, disease modeling, and precision medicine. Given the clinical and basic science evidence supporting an association between the SVZ and GBM, an organoid model that accurately resembles the SVZ would be extremely valuable for the study of GBM. In the basic research sense, such a model may provide a comprehensive picture of gliomagenesis from driver mutations to tumor formation, unlike any other model. Biobanking is critical to improving standardization of study, allowing for research on tumors from patients with naturally developed tumors. Ideally, this model should reliably mimic both structural and cytoarchitectural components of the human SVZ to allow for analysis of the involvement of various niche components in GBM formation and maintenance. Heterogeneity in organoids permits additional factors such as immunologic response to be incorporated to judge their influence on the tumor within the simulated environment (260). Using an SVZ model, therapeutic strategies can be tailored to and tested on tumors in the early stages of development. Organoid models have the advantage of being created using iPSCs from a patient in just a few weeks to allow for the personalization of the treatment regimens for a tumor with specific mutations (261). The SVZ, in particular, is important to study in this context as this is where the earliest mutations are hypothesized to occur meaning this could be the earliest therapeutic target.

**Figure 3:**
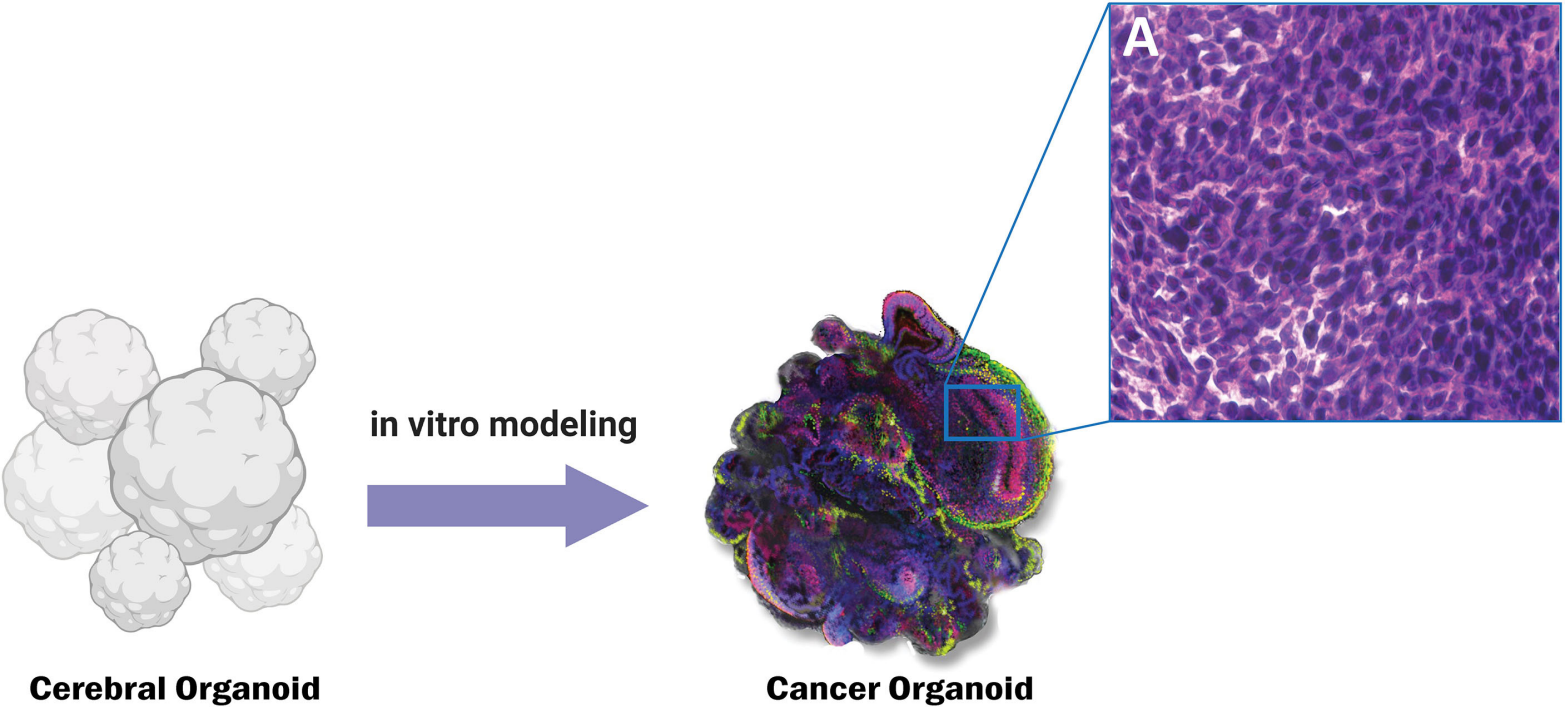
Genetically engineered cancer organoids show histopathological similarity to CNS tumors such as Glioblastoma. Panel A shows the characteristic histopathological features of cancerous tissue such as increased nuclear to cytoplasmic ratio.

Current regional organoid models for the SVZ have some difficulties for GBM research purposes. The Qian et al. (262) model includes SVZ specific cells but lacks non-neurally differentiated structures important to GBM pathology like vasculature and meninges. Lack of vasculature severely limits the size of the organoid. Hypoxia and the absence of nutrients for cells more than 300-500 μm from the surface result in a necrotic core. Due to size limitations, this model only mirrors the human fetal cerebral cortex up to the second trimester, rendering it ineffective for studying tumor pathology in adults (262). Linkous et al. (252) created a model that includes SVZ zone markers, but for the developing brain only. Additionally, no structural or cytoarchitectural analysis of the region confirms an accurately simulated human SVZ (252). Other cerebral organoid models do not demonstrate the presence of a subventricular zone-like region altogether (235, 236, 250, 251, 253, 263). A model that accurately recapitulates the structure, cytoarchitecture, and molecular patterns of the SVZ is necessary for a more comprehensive understanding of GBM initiation and recurrence (**Figure 3**).

## Final Remarks/Conclusion

The recent evidence is in support of NSCs in the SVZ as the cells of origin of GBM, however, the astrocyte dedifferentiation hypothesis has not been rejected and in fact, gliomagenesis can certainly be a combination of oncogenic differentiation and dedifferentiation events. In addition to the site of origin, the SVZ may be involved in the recurrence of GBM as evidenced by improved PFS with radiation therapy to the ipsilateral SVZ (217). Modeling of this region could provide great insight into the pathology of GBM. Such a model could enable therapeutic testing *in vivo*, allowing for the creation of an individualized treatment profile specifically targeting the culprit cells for recurrence (264). Organoid modeling is an all-humanoid and 3D system, an intriguing adjunct model system for brain cancers as opposed to xenotransplantation in animal models and 2D cultures (37). In this review we discuss the role of the subventricular zone in glioblastoma genesis, maintenance, and modeling. We also pointed out the potential impact of introducing novel iPSC-based cerebral organoid SVZ models in the study of gliomagenesis. This is an exciting field of research and may lead to a more personalized approach since iPSC-based models can readily be patient-tailored. It is certain that the SVZ in general and particularly its role in cancer is not entirely understood to date; and it will remain of great interest across various fields of study such as neuroscience, neurodegeneration, and cancer research.

## Author Contributions

Conception and design: PZ. Interpretation of data: PZ and JB. Drafted the manuscript: JB and AH. Approved the final version to be published: PZ. Agree to be accountable for all aspects of the work in ensuring that questions related to the accuracy or integrity of any part of the work are appropriately investigated and resolved: PZ.

## Funding

UPMC University of Pittsburgh medical center startup funds.

## Conflict of Interest

The authors declare that the research was conducted in the absence of any commercial or financial relationships that could be construed as a potential conflict of interest.

## Publisher’s Note

All claims expressed in this article are solely those of the authors and do not necessarily represent those of their affiliated organizations, or those of the publisher, the editors and the reviewers. Any product that may be evaluated in this article, or claim that may be made by its manufacturer, is not guaranteed or endorsed by the publisher.
